# Properties of patient-reported outcome measures in individuals following acute whiplash injury

**DOI:** 10.1186/1477-7525-12-38

**Published:** 2014-03-13

**Authors:** Joshua Pink, Stavros Petrou, Esther Williamson, Mark Williams, Sarah E Lamb

**Affiliations:** 1 Warwick Clinical Trials Unit, Division of Health Sciences, Warwick Medical School, University of Warwick, Coventry CV4 7AL, UK; 2 Nuffield Department of Orthopaedics, Rheumatology and Musculoskeletal Sciences, Medical Sciences Division, University of Oxford, Oxford OX3 7HE, UK

**Keywords:** Whiplash, Outcome assessment, Quality of life, Health status

## Abstract

**Background:**

The aim of this study was to assess the acceptability, reliability, validity and responsiveness of the Short-Form Health Survey (SF-12) and its preference-based derivative (SF-6D), the EQ-5D and the Neck Disability Index (NDI) in patients recovering from acute whiplash injury.

**Methods:**

Data from the Managing Injuries of the Neck Trial of 3,851 patients with acute whiplash injury formed the basis of this empirical investigation. The EQ-5D and SF-12 were collected at baseline, and all three outcome measures were then collected at 4 months, 8 months and 12 months post-randomisation. The measures were assessed for their acceptability (response rates), internal consistency, validity (known groups validity and discriminant validity) and their internal and external responsiveness.

**Results:**

Response rates were broadly similar across the measures, with evidence of a floor effect for the NDI and a ceiling effect for the EQ-5D utility measure. All measures had Cronbach’s α statistics of greater than 0.7, indicating acceptable internal consistency. The NDI and EQ-5D utility score correlated more strongly with the physical component scale of the SF-12 than the mental component scale, whilst this was reversed for the SF-6D utility score. The smaller standard deviations in SF-6D utility scores meant there were larger effect sizes for differences in utility score between patients with different injury severity at baseline than for the EQ-5D utility measure. However, the EQ-5D utility measure and NDI were both more responsive to longitudinal changes in health status than the SF-6D.

**Conclusions:**

There was no evidence of differences between the EQ-5D utility measure and NDI in terms of their construct validity, discriminant validity or responsiveness in patients with acute whiplash injury. However, both demonstrated superior responsiveness to longitudinal health changes than the SF-6D.

## Introduction

Whiplash injuries are soft tissue injuries of the neck that result from an acceleration-deceleration energy transfer mechanism. The prevalence of whiplash injuries is high and is increasing worldwide, particularly within developed countries [1]. Within the United Kingdom (UK) alone the incidence of whiplash injuries is suggested to be around 400,000 per year [1], with the Association of British Insurers noting a 25% rise in whiplash claims during 2002–2008 [1]. Approximately 30-50% of people suffering whiplash injuries report chronic symptoms [2], with an annual cost to the UK economy in 2002 of over £3.1 billion, made up primarily of health service costs and productivity losses [3]. Various treatments for whiplash associated disorders have been proposed, including advice, active management consultations and physiotherapy sessions, but there has been a lack of evidence for both the effectiveness and cost-effectiveness of these interventions [4]. The Managing Injuries of the Neck Trial (MINT) was conducted to fill some of the gaps in this evidence base [5].

Patient reported outcome (PRO) instruments can be used to measure the effects of whiplash injuries in terms of health-related quality of life (HRQoL), and measure the benefits of interventions aimed at their prevention or alleviation. However, there is currently a paucity of evidence on the measurement properties of these instruments when completed by individuals with whiplash injuries. Patient-reported outcome measures (PROMs) are increasingly important outputs for randomised controlled trials [6], as they provide a scientifically robust way of reflecting the patient perspective in the assessment process [7]. Moreover, trial-based economic evaluations are often reliant on preference-based PROMs to calculate the HRQoL component of the quality-adjusted life-year (QALY) metric. Health and Care Excellence (NICE) in England and Wales, for economic evaluations [8].

With the increasing need for quantitative assessment of the impact of preventive or treatment interventions, it is important to identify appropriate outcome measures for use in patients with whiplash injuries. Furthermore, these measures should ideally possess properties, such as internal consistency and construct validity, that satisfy broader regulatory and reimbursement requirements [9]. The MINT study included two generic instruments, the Short-Form Health Survey version 1 (SF-12) [10] and the preference-based EQ-5D-3 L [11], and one neck injury specific measure, the Neck Disability Index (NDI) [12]. Generic instruments are designed to be applicable across a range of health conditions and patient populations, and can be useful to detect unexpected outcomes or side-effects of interventions, which may not be picked up by condition specific measures designed to capture the predicted health status changes. Conversely, more narrowly targeted condition specific measures can provide outputs with a greater clinical relevance, and are often associated with an increased responsiveness compared to generic measures [13]. These differing properties have led to the recommendation for the joint use of generic and condition specific measures in clinical trials [7].

This study compares the three different measures listed above, all of which are commonly used in whiplash injury trials, in terms of their acceptability, reliability, validity and responsiveness in patients with whiplash injuries [14,15].

## Methods

### Study population

Data for this study were drawn from MINT, a pragmatic, cluster randomised controlled trial that recruited patients with acute whiplash injury from 15 NHS emergency departments in the UK [5]. To be eligible for inclusion, patients needed to have a whiplash associated disorder of grades I-III [16]. Patients younger than 18 years of age, with a non-transient loss of consciousness, a Glasgow Coma Score of 12 or less, fractures or dislocations of the spine or other bones, requiring inpatient admission or having a severe psychiatric illness, were excluded. The centres were randomised to provide either active management (including *The Whiplash Book*[17]) or usual care. Patients with substantial symptoms persisting beyond 3 weeks were eligible for further individual randomisation to either a single or six physiotherapy sessions. Since in this study we are primarily interested in the properties of the outcome measures used within MINT, rather than any evaluation of the interventions in the trial, all MINT participants were included in these analyses, regardless of trial allocation.

Patients who consented to be part of the MINT study at the emergency departments were sent an information letter and questionnaires to complete within three days of attendance, including the SF-12 and EQ-5D health outcome measures. These data, which are used as baseline measurements for the analysis, were returned an average of two weeks post emergency department attendance. Further data was collected by postal questionnaires at 4, 8 and 12 months after the initial emergency department attendance, with SF-12 and EQ-5D data, as well as the NDI, collected at each of these time points. On each follow-up occasion, patients failing to respond within a week were sent a second questionnaire and reminder letter, with those still not responding within a further week called twice over the telephone in an attempt to obtain the core MINT outcome measures (NDI and EQ-5D).

### Data collection instruments

The SF-12 consists of 12 questions with a one week recall period measuring various aspects of physical and mental health, from which three summary scores can be extracted. The Physical Component Summary Score (PCS) and Mental Component Summary Score (MCS) are both standardised to have a mean of 50 and a standard deviation of 10 [18], whilst a six dimension health-state classification based on the SF-12, called the SF-6D, can also be constructed, containing 7,500 potential health states with utility values, calculated using the standard gamble technique, ranging from 0.345 to 1 [19].

The EQ-5D contains six items, and asks people about their health state on the day they complete the questionnaire. The first five items ask the respondent to describe their mobility, self-care, usual activities, pain/discomfort and anxiety/depression in the form of a health state classification system. Responses to each of these five dimensions are divided into three ordinal levels coded: (1) no problems; (2) some or moderate problems; and (3) severe or extreme problems. A total of 243 (3^5^) health states are generated by the EQ-5D descriptive system. Responses to the five item descriptive system can be converted into utility scores using a UK specific tariff [20], calculated from a time trade-off study, taking values between −0.59 and 1, with 1 corresponding to “perfect health” and 0 representing a health state considered to be equivalent to death [11]. The sixth item of the EQ-5D consists of a visual analogue scale (VAS) and asks people to rate their current overall health on a scale from 0 (the worst health state they can imagine) to 100 (the best health state they can imagine).

The NDI consists of 10 questions measuring neck pain-related activity restrictions, with each item scored on a scale from 0 (no restriction) to 5 (severe restriction). These scores are then summed to give a total score ranging from 0 to 50 then doubled to scale to a score from 0 to 100. Vernon et al. have published a categorisation for these NDI scores, with a total score of 4 or less corresponding to no disability, 5–14 mild disability, 15–24 moderate disability, 25–34 severe disability and greater than 34 complete disability [12]. These categorisations are now a commonly used approach in analyses making use of the NDI [21].

### Statistical analysis

The MINT study contained no specific questions looking at the acceptability of the different outcome measures used, and no information was collected on the reasons for missing data where a particular questionnaire was returned, but not all items within it were completed. Therefore, the acceptability of the measures (EQ-5D(utility), EQ-5D (VAS), SF-6D, SF-12(PCS), SF-12(MCS) and NDI) was assessed by looking at the response rates to each measure at each time point of assessment, as well as the individual item and dimension completion rates [22]. Whilst this will provide less information than would have been available if patients had been directly questioned [23], there is evidence of a link between response rates and acceptability of a questionnaire to respondents [24,25].

The internal consistency of the EQ-5D(utility), SF-6D and NDI, that is, the extent to which multiple items in each scale measure the same underlying concept, was assessed by calculating Cronbach’s α coefficients [26]. This has also often been used, in the absence of any better method, as a proxy for the overall reliability of the instrument, that is, the stability and consistency of the concept being measured. Whilst there are questions as to how relevant a concept internal consistency is for preference-based measures [22], an established convention has been to deem a score of ≥0.70 to be sufficient for use in research, and a score of ≥0.90 for broader use in routine clinical practice [25]. It would be expected that the NDI, since it covers a narrower range of outcomes than the EQ-5D and SF-6D, would have the highest Cronbach’s α.

The construct validity of the measures was assessed in terms of both known groups validity and discriminant validity [14]. In known groups validity, we take pre-specified groups where we would expect there to be a difference in health status, and thus instrument scores. The different scores between groups for alternative measures can then be compared to see if there is a pattern in the sensitivity to these expected differences [26]. We classified patients according to whiplash associated disorder (WAD) grades at baseline, and performed independent samples t-tests for differences in baseline EQ-5D(utility), EQ-5D(VAS), SF-6D, SF-12(PCS) and SF-12(MCS) scores, or differences in NDI at 4 months, the latter measure not having been included at baseline. The magnitude of these differences was compared by calculating effect sizes, i.e. the mean difference between the WAD grade groups (either WAD grade 1 versus WAD grade 2 or WAD grade 1 versus WAD grade 3) standardised by dividing by the pooled standard deviation of the two groups. This standardisation allows for the unbiased comparison of measures with differing scales [27]. A standard, if again largely arbitrary, classification system devised by Cohen regards an effect size of 0.20 as small, 0.50 as moderate and 0.80 or greater as large [27].

Discriminant validity, the extent to which different instruments with overlapping constructs converge or diverge, was tested by calculating Pearson’s correlation coefficients and Spearman’s rank correlation coefficients between each of the summary scores of the EQ-5D, SF-6D and NDI. A higher correlation between one of the two utility measures and the NDI cannot interpreted as evidence of superiority in psychometric terms over the other utility measure (the NDI cannot be regarded as a gold standard and generic, preference based measures are not intended to measure the same constructs as condition specific measures [22]). Nevertheless, it could be regarded as evidence of a greater degree of construct overlap between that utility measure and the NDI. Spearman’s correlations were also calculated for the individual items within and between each measurement, with the assumption being that similar dimensions in different measures should correlate more highly than different dimensions within the same measure.

We assessed both the internal and external responsiveness of the EQ-5D(utility), SF-6D and NDI. The internal responsiveness of a measure represents its ability to detect changes over a specified timeframe [28]. We calculated effect sizes (the mean change in measure over time divided by the standard deviation pooled across the two time points) and standardised response means (these differ from effect sizes as they are standardised by dividing by the standard deviation of the difference between the measures at the two time points) for the changes in each measure over time, together with the associated 95% confidence intervals [29]. We also calculated the proportion of patients in floor (the lowest possible) or ceiling (the highest possible) health states at each time point, as a high proportion of individuals at one end of the scale can indicate a lack of specificity in that region as well as a lack of responsiveness to change.

External responsiveness considers whether the changes registered by a measure over time correspond to those expected based on an external reference measure of health [28]. We made use of two different reference measures to measure two different aspects of responsiveness, responsiveness to self-reported changes in neck injury status and responsiveness to changes in NDI score. Firstly, we used a question asked to the patient at each follow-up point as to whether their neck injury was much worse, worse, the same, better or much better than at the time of completion of the previous questionnaire. Mean differences, standardised response means and effect sizes were calculated for the changes in outcome measures for patients in each of these self-reported groups. A more responsive measure should show larger differences between the self-reported groups. Secondly, we used various categorisations of the NDI as our reference measure, to see which of the utility measures, EQ-5D or SF-6D, better captured changes in neck disability. The NDI categorisations used were: change in NDI score between 4 months and 12 months, change in Vernon category between 4 months and 12 months, 4 month Vernon categories, 12 month Vernon categories and finally a categorisation of patient outcome trajectories defined by Sterling et al., where neck injuries are classed as either mild, moderate or chronic-severe [30]. These trajectories had been constructed from a previous data set of 155 individuals monitored for one year post whiplash injury [30].

Analysis was carried out using SPSS version 21 [31] and R version 2.15.1 [32].

## Results

3,851 individuals were randomised in MINT, 1,006 of whom were complete responders, that is, they returned the SF-12 and EQ-5D measures at baseline, 4 months, 8 months and 12 months and the NDI at 4 months, 8 months and 12 months. The baseline characteristics of the whole population and of complete versus non-compete responders are given in Table 1. There were significant differences (at the 95% level) between responders and non-responders in all the characteristics examined, with the exception of WAD grade at baseline [5]. Complete responders tended to be older, and were more likely to be female, with lower pain intensity at baseline. Response rates were also higher from people randomised to the control group (at either randomisation) than those assigned to the MINT interventions.

**Table 1:** Baseline characteristics of the MINT study population

	**Whole population (n = 3,851)**	**Complete responders (n = 1,006)**	**Non-complete responders (n = 2,845)**
Age: mean (SD)	36.98 (13.42)	37.91 (12.44)	36.65 (13.74)
Sex:			
% male	1661/3851 (43.1%)	385/1006 (38.3%)	1276/2845 (44.9%)
% female	2133/3851 (55.4%)	614/1006 (61.0%)	1519/2845 (53.4%)
Treatment group step 1:			
% usual care	1598/3851 (41.5%)	452/1006 (44.9%)	1146/2845 (40.3%)
% active management	2253/3851 (58.5%)	554/1006 (55.1%)	1699/2845 (59.7%)
Treatment group step 2*:			
% advice	287/574 (50%)	103/198 (55.1%)	184/376 (48.9%)
% physiotherapy	287/574 (50%)	95/198 (44.9%)	192/376 (51.1%)
Initial pain intensity: mean (SD)	5.13 (1.89)	4.79 (1.78)	5.25 (1.92)
WAD grade at presentation:			
% grade 1	2088/3851 (54.2%)	557/1006 (55.4%)	1531/2845 (53.8%)
% grade 2	1659/3851 (43.1%)	424/1006 (42.1%)	1235/2845 (43.4%)
% grade 3	104/3851 (2.7%)	25/1006 (2.5%)	79/2845 (2.8%)

The percentages may not add up to 100% due to missing data. *The denominators are lower here as not all participants were randomised at step 2.

### Acceptability

Table 2 shows the response rates (assessed in terms of complete responses to all relevant questions) for each of the measures. Baseline response rates varied from 78.6% (the SF-6D subset of the SF-12) to 89.1% (EQ-5D utility), whilst response rates at the end of the follow-up period varied from 50.4%-69.2%. There were very low rates (<2%) of partial completion (defined as failure to complete at least one item) across measures and follow-up points, with individuals tending to either complete the whole measure or not respond at all.

**Table 2:** Response rates to each measure over time; % of non-missing data by questionnaire and time point (sample size of 3,851 in all cases)

	**Baseline**	**4 months**	**8 months**	**12 months**	**Complete responders (all time points)**
EQ-5D (utility)	89.1%	78.4%	70.2%	69.2%	49.7%
EQ-5D (VAS)	87.3%	76.5%	68.9%	67.7%	46.9%
SF-6D*	78.6%	63.7%	55.5%	51.3%	30.3%
SF-12 (PCS)	79.9%	62.5%	55.1%	50.4%	30.0%
SF-12 (MCS)	79.9%	62.5%	55.1%	50.4%	30.0%
NDI	N/A	76.9%	69.4%	68.5%	51.9%

*The SF-6D was regarded as complete if there were sufficient items to calculate a utility score.

### Reliability

Cronbach’s alpha scores were 0.790, 0.871 and 0.922 for the EQ-5D(utility), SF-6D and NDI, respectively, all above the threshold of 0.70 recommended for broader use in clinical research. The Cronbach’s alpha score for the NDI was also above the 0.90 cut-off recommended for use in routine clinical practice. A higher value would be expected for this measure due to the narrower range of impacts it aims to capture.

### Validity

Descriptive statistics for each of the measures at baseline are shown in Table 3 (with the exception of the NDI for which descriptive statistics at the 4 month follow-up are presented). There is some evidence of a floor effect (scores of 0) with the NDI and a ceiling effect (scores of 1) with the EQ-5D utility measure, but no measure has more than 11.5% of scores at either extreme of a scale. The results of tests of known groups validity summarised in Table 4 show that there were differences in scores for all measures at baseline between pre-specified WAD groups (1 versus 2 and 1 versus 3). All differences are statistically significant at the 5% level between WAD grades 1 and 2. The small number of individuals in WAD grade 3 (n = 104) meant that only the EQ-5D(VAS), SF-6D and SF-12(MCS) differences are significant between WAD grades 1 and 3, despite the magnitude of the differences being larger in all cases than those observed for WAD grades 1 versus 2. The SF-6D had larger effect sizes than the EQ-5D utility measure across both comparisons (grade 1 versus grade 2 and grade 1 versus grade 3), though they both fall into the small-moderate range as defined by the Cohen classifications. Specifically, the effect sizes for the EQ-5D and SF-6D, respectively, were 0.310 and 0.364 between grades 1 and 2, and 0.353 and 0.496 between grades 1 and 3.

**Table 3:** Descriptive statistics for measures (baseline data with the exception of the NDI (4 months))

	**Outcome range**	**N**	**Mean (SD)**	**Median**	**Floor**	**Ceiling**
EQ-5D (utility)	−0.594 to 1	3430	0.587 (0.298)	0.689	0.1%	8.3%
EQ-5D (VAS)	0 to 100	3361	63.67 (19.96)	65.0	0.1%	0.8%
SF-6D	0.41 to 1	3027	0.647 (0.136)	0.615	0%	0.7%
SF-12 (PCS)*	15.42 to 65.92	3076	40.26 (8.98)	39.16	0%	0%
SF-12 (MCS)*	9.51 to 68.21	3076	40.80 (12.80)	40.25	0%	0%
NDI	0 to 96	2963	21.03 (17.45)	18.0	11.5%	0%

*The PCS and MCS are standardised to have a mean of 50 and a SD of 10 in the general population.

**Table 4:** Known groups validity effect sizes (baseline data with the exception of the NDI (4 months))

	**WAD grade 1**	**WAD grade 2**	**Difference (95% confidence interval)**	**Effect size for group 1 vs group 2 (95% confidence interval)**
EQ-5D (utility)	0.684 (0.235)	0.606 (0.272)	0.078 (0.045, 0.110)	0.310 (0.184, 0.437)
EQ-5D (VAS)	68.84 (18.23)	63.80 (18.55)	5.04 (2.72, 7.36)	0.274 (0.147, 0.401)
SF-6D	0.680 (0.143)	0.630 (0.130)	0.050 (0.032, 0.067)	0.364 (0.237, 0.491)
SF-12 (PCS)	42.41 (9.24)	39.41 (8.83)	3.01 (1.84, 4.17)	0.331 (0.204, 0.458)
SF-12 (MCS)	44.04 (11.97)	41.42 (12.15)	2.62 (1.07, 4.17)	0.217 (0.090, 0.344)
NDI	15.64 (14.21)	20.03 (15.22)	−4.39 (−6.24, −2.54)	−0.300 (−0.427, −0.173)
	**WAD grade 1**	**WAD grade 3**	**Difference (95% confidence interval)**	**Effect size for group 1 vs group 3 (95% confidence interval)**
EQ-5D (utility)	0.684 (0.235)	0.600 (0.300)	0.083 (−0.042, 0.209)	0.353 (−0.048, 0.754)
EQ-5D (VAS)	68.84 (18.23)	59.60 (20.92)	9.24 (1.88, 16.61)	0.508 (0.106, 0.910)
SF-6D	0.680 (0.143)	0.609 (0.149)	0.070 (0.129, 0.128)	0.496 (0.094, 0.897)
SF-12 (PCS)	42.41 (9.24)	39.14 (9.87)	3.28 (−0.45, 7.00)	0.353 (−0.048, 0.754)
SF-12 (MCS)	44.04 (11.97)	38.48 (12.57)	5.56 (0.74, 10.38)	0.463 (0.061, 0.864)
NDI	15.64 (14.21)	22.89 (19.30)	−7.25 (−15.29, 0.79)	−0.503 (−0.905, −0.101)

Table 5 shows the correlation coefficients between the various summary measures, with all correlations statistically significant at the 1% level. The SF-6D correlates more strongly with the mental component scale (rather than the physical component scale) of the SF-12, whilst the EQ-5D (both utility and VAS measures) and NDI correlate more strongly with the physical component scale, with the NDI being more strongly correlated with the EQ-5D utility measure than the SF-6D. Individual item correlations followed the expected patterns (i.e. significant positive correlations between worsening health states on all items within and between the SF-6D, EQ-5D utility and NDI measures) with a smallest correlation coefficient of 0.202 (between the self-care dimension from the EQ-5D and the mental health dimension from the SF-6D). Dimensions measuring similar constructs also correlated more highly than others with, as an example, the pain questions on each measure all having correlations of greater than 0.615 between one another.

**Table 5:** Pearson’s (top-right) and Spearman’s (bottom-left) correlations between measures

**Pearson (TR)/Intra-class (BL) correlations**	**EQ-5D (utility)**	**EQ-5D (VAS)**	**SF-6D**	**SF-12 (PCS)**	**SF-12 (MCS)**	**NDI**
EQ-5D (utility)	N/A	0.676	0.691	0.663	0.601	−0.762
EQ-5D (VAS)	0.694	N/A	0.671	0.609	0.610	−0.628
SF-6D	0.774	0.690	N/A	0.683	0.801	−0.714
SF-12 (PCS)	0.732	0.616	0.672	N/A	0.298	−0.771
SF-12 (MCS)	0.621	0.612	0.807	0.299	N/A	−0.504
NDI	−0.792	−0.600	−0.693	−0.759	−0.464	N/A

Time points were combined and missing data excluded pairwise for each comparison.

*p values were less than 0.001 for all correlations in this table.

### Responsiveness

Tables 6, 7 and 8 display measures of the responsiveness of the EQ-5D(utility), SF-6D and NDI, respectively, using self-reported change in neck injury as the referent. Tables 9 and 10 display similar results for the EQ-5D(utility) and SF-6D, but using the NDI as the referent. In Table 6, when data were combined across all possible time points of comparison, there were statistically significant differences in changes in EQ-5D utility scores between alternative categories of self-reported neck injury, ranging from a change of −0.2961 for patients reporting their injury had got much worse to a change of 0.0955 for those reporting it had got much better. This was also the case for the SF-6D (Table 7) with the exception of the difference in change in utility score between the better (0.0643) and much better (0.0613) self-reported categories, which went in the reverse order to that which would be expected. Effect sizes and standardised response means were consistently larger for the EQ-5D(utility) than for the SF-6D (by an average of 49.8%), and were also consistently ordered across self-reported categories for the EQ-5D utility measure, which was not the case for the SF-6D. There was no consistent pattern of differences between the EQ-5D(utility) and NDI, with effect sizes differing by a smaller average of 16.9%. Furthermore, there was a consistent pattern across all three measures for individuals reporting that their neck injury was the same as 4 months previously, with all showing (when time points were pooled) a small improvement in score.

**Table 6:** Responsiveness of the EQ-5D over time, anchored by self-reported change in neck injury

	**T**_**0**_	**T**_**1**_	**∆**	**SRM (95% CI)**	**ES (95% CI)**	**n**
**EQ-5D utility baseline to 4 months with anchor of neck injury change baseline to 4 months**						
Much worse	0.7363	0.3253	−0.4110	−1.0516 (−2.4740, 0.4861)	−1.9282 (−3.9172, 0.1913)	3
Worse	0.4838	0.5172	0.0334	0.1408 (−0.2908, 0.5690)	0.1077 (−0.4983, 0.7123)	21
Same	0.5731	0.6784	0.1053	0.4001 (0.2303, 0.5686)	0.4059 (0.1730, 0.6381)	145
Better	0.6162	0.7634	0.1473	0.6028 (0.4999, 0.7051)	0.7225 (0.5845, 0.8601)	431
Much better	0.7306	0.8898	0.1592	0.7998 (0.6846, 0.9142)	0.8748 (0.7267, 1.0224)	385
**EQ-5D utility 4 months to 8 months with anchor of neck injury change 4 months to 8 months**						
Much worse	N/A	N/A	N/A	N/A	N/A	0
Worse	0.7302	0.6754	−0.0549	−0.2346 (−0.5145, 0.0476)	−0.2668 (−0.6599, 0.1276)	50
Same	0.8060	0.8102	0.0042	0.0294 (−0.0886, 0.1474)	0.0198 (−0.1471, 0.1866)	276
Better	0.7417	0.7919	0.0502	0.2692 (0.1611, 0.3769)	0.2698 (0.1191, 0.4203)	342
Much better	0.8492	0.9310	0.0818	0.5271 (0.4092, 0.6442)	0.5530 (0.3942, 0.7114)	317
**EQ-5D utility 8 months to 12 months with anchor of neck injury change 8 months to 12 months**						
Much worse	0.6516	0.4704	−0.1812	−0.7190 (−1.6866, 0.3129)	−0.4700 (−1.7162, 0.8041)	5
Worse	0.7499	0.6484	−0.1016	−0.4224 (−0.6872, −0.1543)	−0.4723 (−0.8372, −0.1054)	59
Same	0.8474	0.8460	−0.0014	−0.0089 (−0.1122, 0.0944)	−0.0070 (−0.1531, 0.1391)	360
Better	0.7889	0.8377	0.0489	0.2847 (0.1634, 0.4055)	0.2638 (0.0952, 0.4322)	273
Much better	0.8868	0.9324	0.0456	0.3195 (0.2008, 0.4376)	0.2957 (0.1313, 0.4598)	288
**EQ-5D utility: above three combined**						
Much worse	0.6940	0.3979	−0.2961	−0.9696 (−1.8010, −0.0944)	−0.8376 (−1.8512, 0.2030)	8
Worse	0.6546	0.6136	−0.0410	−0.1728 (−0.3457, 0.0007)	−0.1788 (−0.4222, 0.0650)	130
Same	0.7422	0.7782	0.0360	0.2034 (0.1325, 0.2742)	0.1661 (0.0667, 0.2654)	781
Better	0.7156	0.7977	0.0821	0.3930 (0.3300, 0.4558)	0.4226 (0.3359, 0.5092)	1046
Much better	0.8222	0.9177	0.0955	0.5605 (0.4934, 0.6274)	0.5815 (0.4915, 0.6714)	990

**Table 7:** Responsiveness of the SF-6D over time, anchored by self-reported change in neck injury

	**T**_**0**_	**T**_**1**_	**∆**	**SRM (95% CI)**	**ES (95% CI)**	**n**
**SF-6D baseline to 4 months with anchor of neck injury change baseline to 4 months**						
Much worse	0.6800	0.6963	0.0163	0.1023 (−1.0446, 1.2261)	0.2037 (−1.4145, 1.7977)	3
Worse	0.6032	0.6165	0.0132	0.0836 (−0.3459, 0.5110)	0.1063 (−0.4997, 0.7109)	21
Same	0.6335	0.7019	0.0683	0.5939 (0.4164, 0.7696)	0.4961 (0.2620, 0.7294)	145
Better	0.6252	0.7628	0.1376	0.9996 (0.8836, 1.1148)	1.1166 (0.9728, 1.2599)	431
Much better	0.7085	0.8343	0.1257	0.8401 (0.7235, 0.9559)	0.9636 (0.8141, 1.1126)	385
**SF-6D 4 months to 8 months with anchor of neck injury change 4 months to 8 months**						
Much worse	N/A	N/A	N/A	N/A	N/A	0
Worse	0.7413	0.6979	−0.0434	−0.3673 (−0.6521, −0.0790)	0.1433 (−0.2496, 0.5354)	50
Same	0.7687	0.7836	0.0149	0.1355 (0.0169, 0.2539)	0.1401 (−0.0270, 0.3071)	276
Better	0.7498	0.7872	0.0374	0.3429 (0.2336, 0.4517)	0.1243 (−0.0258, 0.2743)	342
Much better	0.8237	0.8619	0.0382	0.3385 (0.2250, 0.4514)	0.1129 (−0.0429, 0.2687	317
**SF-6D 8 months to 12 months with anchor of neck injury change 8 months to 12 months**						
Much worse	0.6425	0.6035	−0.0390	−0.2883 (−1.1694, 0.6256)	−0.3175 (−1.5569, 0.9411)	5
Worse	0.7387	0.6905	−0.0482	−0.3272 (−0.5879, −0.0639)	−0.3707 (−0.7339,-0.0059)	59
Same	0.8078	0.8086	0.0008	0.0080 (−0.0953, 0.1113)	0.0059 (−0.1402, 0.1520)	360
Better	0.7804	0.7983	0.0178	0.1757 (0.0560, 0.2951)	0.1461 (−0.0219, 0.3140)	273
Much better	0.8435	0.8634	0.0199	0.2027 (0.0859, 0.3192)	0.1802 (0.0165, 0.3438)	288
**SF-6D: above three combined**						
Much worse	0.6613	0.6499	−0.0114	−0.0789 (−0.7703, 0.6180)	−0.1028 (−1.0817, 0.8797)	8
Worse	0.6944	0.6683	−0.0261	−0.1885 (−0.3616, −0.0147)	−0.1941 (−0.4376, 0.0498)	130
Same	0.7367	0.7647	0.0280	0.2627 (0.1913, 0.3340)	0.2039 (0.1044, 0.3033)	781
Better	0.7185	0.7828	0.0643	0.5360 (0.4711, 0.6007)	0.5215 (0.4343, 0.6086)	1046
Much better	0.7919	0.8532	0.0613	0.4907 (0.4247, 0.5565)	0.5124 (0.4228, 0.6019)	990

**Table 8:** Responsiveness of the NDI over time, anchored by self-reported change in neck injury

	**T**_**0**_	**T**_**1**_	**∆**	**SRM (95% CI)**	**ES (95% CI)**	**n**
**NDI 4 months to 8 months with anchor of neck injury change 4 months to 8 months**						
Much worse	N/A	N/A	N/A	N/A	N/A	0
Worse	23.764	29.804	6.040	0.5816 (0.2790, 0.8791)	0.4163 (0.0190, 0.8116)	50
Same	16.031	15.753	−0.560	−0.0735 (−0.1916, 0.0447)	−0.0336 (−0.2004, 0.1333)	276
Better	22.030	16.853	−5.446	−0.5521 (−0.6655, −0.4380)	−0.4129 (−0.5642, −0.2613)	342
Much better	11.212	4.775	−6.439	−0.7657 (−0.8904, −0.6400)	−0.7691 (−0.9302, −0.6075)	317
**NDI 8 months to 12 months with anchor of neck injury change 8 months to 12 months**						
Much worse	34.800	47.600	12.800	0.9257 (−0.1823, 1.9635)	0.5261 (−0.7546, 1.7759)	5
Worse	21.653	27.792	6.139	0.6321 (0.3500, 0.9096)	0.4117 (0.0461, 0.7755)	59
Same	13.269	13.155	−0.114	−0.0179 (−0.1212, 0.0854)	−0.0075 (−0.1536, 0.1386)	360
Better	16.921	12.897	−4.024	−0.5046 (−0.6302, −0.3782)	−0.3391 (−0.5079, −0.1700)	273
Much better	7.873	4.192	−3.681	−0.6068 (−0.7320, −0.4807)	−0.4831 (−0.6486, −0.3172)	288
**NDI: above two combined**						
Much worse	34.800	47.600	12.800	0.9257 (−0.1823, 1.9635)	0.5261 (−0.7546, 1.7759)	5
Worse	22.709	28.799	6.090	0.6074 (0.4018, 0.8106)	0.4134 (0.1446, 0.6813)	109
Same	14.650	14.454	−0.337	−0.0486 (−0.1263, 0.0292)	−0.0213 (−0.1312, 0.0886)	636
Better	19.476	14.875	−4.735	−0.5218 (−0.6059, −0.4374)	−0.3752 (−0.5879, −0.3752)	615
Much better	9.543	4.484	−5.060	−0.6850 (−0.7733, −0.5962)	−0.6307 (−0.7460, −0.5151)	605

**Table 9:** Responsiveness of the EQ-5D to changes between 4 and 12 months, anchored by NDI classifications

	**T**_**0**_	**T**_**1**_	**∆**	**SRM (95% CI)**	**ES (95% CI)**	**n**
**EQ-5D utility changes based on NDI score changes**						
Decrease	0.7563	0.8639	0.1076	0.6034 (0.5211, 0.6854)	0.5890 (0.4798, 0.6980)	673
No change	0.9215	0.9267	0.0053	0.0465 (−0.0958, 0.1887)	0.0381 (−0.1630, 0.2392)	190
Increase	0.7872	0.7526	−0.3145	−1.7008 (−1.9078, −1.4921)	−1.4186 (−1.6280, −1.2078)	218
**EQ-5D utility changes based on Vernon category changes**						
Decrease	0.7121	0.8706	0.1585	0.8649 (0.7452, 0.9838)	0.8734 (0.7225, 1.0238)	371
No change	0.8412	0.8662	0.0250	0.1780 (0.0993, 0.2566)	0.1420 (0.0315, 0.2525)	631
Increase	0.7682	0.6588	−0.1094	−0.4952 (−0.7275, −0.2600)	−0.4259 (−0.7406, −0.1098)	79
**EQ-5D utility changes based on 4 month Vernon categories**						
None	0.9431	0.9392	0.0061	0.0505 (−0.0509, 0.1519)	0.0545 (−0.0889, 0.1978)	374
Mild	0.7682	0.8570	0.0888	0.5061 (0.4107, 0.6010)	0.6156 (0.4858, 0.7451)	479
Moderate	0.6375	0.7174	0.0798	0.3444 (0.1972, 0.4907)	0.4195 (0.2154, 0.6231)	189
Severe	0.3879	0.5329	0.1451	0.4987 (0.1488, 0.8423)	0.4964 (0.0255, 0.9639)	36
Complete	0.1783	0.4307	0.2523	0.9407 (−0.5358, 2.2996)	1.5170 (−0.4398, 3.3493)	3
**EQ-5D utility changes based on 12 month Vernon categories**						
None	0.8712	0.9563	0.0851	0.5622 (0.4752, 0.6488)	0.6759 (0.5584, 0.7931)	590
Mild	0.7364	0.7848	0.0484	0.2643 (0.1561, 0.3701)	0.3179 (0.1695, 0.4660)	354
Moderate	0.6325	0.6218	−0.0106	−0.0442 (−0.2239, 0.1357)	−0.0489 (−0.3030, 0.2053)	119
Severe	0.3964	0.3452	−0.0512	−0.1335 (−0.6573, 0.3953)	−0.1995 (−0.9404, 0.5452)	14
Complete	0.0300	0.1683	0.1383	1.4385 (−0.0687, 2.8662)	0.5549 (−0.8860, 1.9529)	4
**EQ-5D utility changes based on stirling trajectories**						
Mild	0.8488	0.9090	0.0602	0.3913 (0.3201, 0.4623)	0.4292 (0.3311, 0.5272)	818
Moderate	0.6383	0.7819	0.1436	0.6822 (0.4871, 0.8751)	0.8367 (0.5783, 1.0935)	126
Chronic-severe	0.5908	0.5803	−0.0104	−0.0408 (−0.2082, 0.1268)	−0.0418 (−0.2786, 0.1951)	137

**Table 10:** Responsiveness of the SF-6D to changes between 4 and 12 months, anchored by NDI classifications

	**T**_**0**_	**T**_**1**_	**∆**	**SRM (95% CI)**	**ES (95% CI)**	**n**
**SF-6D changes based on NDI score changes**						
Decrease	0.7592	0.8200	0.0608	0.5328 90.4519, 0.6134)	0.4813 (0.3728, 0.5896)	673
No change	0.8523	0.8674	0.0152	0.1884 (0.0447, 0.3316)	0.1386 (−0.0628, 0.3398)	190
Increase	0.7656	0.7406	−0.0250	−0.2221 (−0.3562, −0.0875)	−0.1854 (−0.3734, 0.0028)	218
**SF-6D changes based on Vernon category changes**						
Decrease	0.7412	0.8189	0.0777	0.6508 (0.5384, 0.7625)	0.6096 (0.4622, 0.7566)	371
No change	0.8019	0.8219	0.0201	0.1975 (0.1186, 0.2762)	0.1593 (0.0487, 0.2698)	631
Increase	0.7444	0.7052	−0.0392	−0.3482 (−0.5743, −0.1200)	−0.2858 (−0.5988, 0.0281)	79
**SF-6D changes based on 4 month Vernon categories**						
None	0.8594	0.8730	0.0136	0.1482 (0.0462, 0.2500)	0.1445 (0.0009, 0.2880)	374
Mild	0.7827	0.8161	0.0344	0.2878 (0.1963, 0.3790)	0.2922 (0.1648, 0.4194)	479
Moderate	0.6437	0.7202	0.0766	0.5699 (0.4155, 0.7230)	0.6665 (0.4589, 0.8732)	189
Severe	0.5638	0.6408	0.0770	0.6503 (0.2863, 1.0067)	0.7700 (0.2882, 1.2466)	36
Complete	0.5018	0.5150	0.0132	0.1982 (−0.9695, 1.3221)	0.2469 (−1.3765, 1.8411)	3
**SF-6D changes based on 12 month Vernon categories**						
None	0.8333	0.8733	0.0400	0.3844 (0.3006, 0.4679)	0.4019 (0.2866, 0.5171)	590
Mild	0.7378	0.7768	0.0389	0.3096 (0.2027, 0.4160)	0.3293 (0.1809, 0.4775)	354
Moderate	0.6490	0.6552	0.0062	0.0497 (−0.1302, 0.2294)	0.0544 (−0.1998, 0.3085)	119
Severe	0.5615	0.5666	0.0051	0.0538 (−0.4714, 0.5770)	0.0836 (−0.6583, 0.8239)	14
Complete	0.4669	0.5034	0.0365	1.9333 (0.1445, 3.6664)	2.3982 (0.4331, 4.2670)	4
**SF-6D changes based on stirling trajectories**						
Mild	0.8197	0.8485	0.0288	0.2746 (0.2047, 0.3443)	0.2689 (0.1715, 0.3662)	818
Moderate	0.6530	0.7633	0.1103	0.8241 (0.6206, 1.0251)	0.9941 (0.7314, 1.2551)	126
Chronic-severe	0.6348	0.6417	0.0070	0.0583 (−0.1094, 0.2258)	0.0612 (−0.1757, 0.2980)	137

For the analyses using NDI categorisations as reference categories, summarised in Tables 9 and 10, both the EQ-5D(utility) and the SF-6D were consistently more responsive when a longitudinal reference category was used, that is, the referent was delineated as a change in a measure rather than a value at a given time point. Whilst there was considerable variability between effect sizes and standardised response means based on the reference category used, the EQ-5D utility measure again came out as consistently more responsive than the SF-6D (effect sizes and standardised response means were respectively, on average, 22.5% and 13.1% higher for the EQ-5D utility measure than for the SF-6D).

## Discussion

The intention of this study was to compare the properties of different patient-reported outcome measures that have been used following acute whiplash injury. The results show significant variation between instrument properties (known groups discrimination, responsiveness etc.) when used in this population.

When comparing different patient-reported outcome measures, there are a number of specific difficulties with interpretation that it is important to note [33]. First, the underlying concepts and domains of health measured will not be the same with, in our case, the EQ-5D and SF-12 being generic health measures whilst the NDI is neck-injury specific. They also relate to different time periods, with the EQ-5D asking specifically about an individual’s health ‘today’, the version of the SF-12 in MINT using a one-week recall period and the NDI asking about current capabilities without specifying a time frame. Scales and the outcome space of possible answers also differ, a problem that can be partially, though not entirely, addressed by standardisation (i.e. effect sizes or standardised response means), and the directions of values for better health are not always the same, with higher NDI scores corresponding to worse health, the reverse being the case for the other measures. When considering effect sizes and standardised response means, it is important to remember that differences between measures can be driven by differences in magnitude, differences in variability or both, which can make interpretations of these statistics more difficult.

With all these provisos taken into account, there was little evidence of differences in response or completion rates between the different measures. Whilst there were higher response rates to the EQ-5D and NDI as opposed to the SF-12 this can be explained, at least in part, by the follow-up methodology within MINT (missing EQ-5D and NDI questionnaires were chased by postal reminders and telephone contacts, whilst missing SF-12 questionnaires were chased by postal reminders only). There were no meaningful differences if response rates were compared prior to the additional telephone contacts. In the postal questionnaires, the NDI was presented as the first question, the SF-12 the second and the EQ-5D the third, meaning that if questionnaire length is leading to participant fatigue and subsequent non-completion, we would expect higher response rates to the NDI than the EQ-5D. However, we in fact find the reverse pattern, with very slightly (though non-significantly) higher response rates to the EQ-5D.

The EQ-5D(utility) and NDI both appear to be more responsive to longitudinal changes in health status than the SF-6D and give results consistent with the expected trend of deteriorating health status resulting in lower utility values (EQ-5D) or increasing scores (NDI), whilst the SF-6D does not. The EQ-5D(utility) correlates more strongly with the NDI than the SF-6D does, perhaps implying a higher level of construct overlap, and both the EQ-5D(utility) and NDI correlate more strongly with the PCS of the SF-12 than the MCS, the opposite of the case for the SF-6D. The low level of correlation between the MCS and PCS scales of the SF-12 (0.298, the lowest between any two measures) indicates that these constructs are indeed non-overlapping to a considerable extent.

In contrast, the SF-6D produces larger effect sizes for differences in injury severity (WAD grade) than the EQ-5D utility measure at a fixed time point. This may, however, be driven by the lower standard deviation for SF-6D values (in turn driven, at least in part, by the lower possible range of outcome values) rather than larger differences between the groups themselves. Indeed, the differences in mean utility values between the groups are again larger for the EQ-5D utility measure than for the SF-6D. The SF-6D does have the advantage of showing no discernible floor or ceiling effects, in contrast to both the NDI and EQ-5D utility measure. The EQ-5D-5 L, a modification of the standard EQ-5D that provides five response levels in each dimension, should help to address this issue, but it is not yet in widespread use [34].

In order to try and understand the reasons for these differences, it is important to consider both the descriptive systems of the instruments and, for preference based measures, the valuation methods [22], and there are marked differences between the SF-6D and EQ-5D in both these areas [35]. The SF-6D has more levels than the EQ-5D, and is more concentrated on milder health problems, with the worst states in the SF-6D descriptive system arguably less severe than those in the EQ-5D descriptive system [35,36]. There is evidence that the SF-6D is better able to detect small changes in health, and is more sensitive to changes in health status at the top end of the distribution, whilst the EQ-5D is more sensitive to health change in individuals with poor baseline health [35]. There are also differences in the valuation method, with the EQ-5D valued using the time-trade off approach and the SF-6D valued using the standard gamble approach. There is empirical evidence that the time-trade off approach results in higher values for milder states and lower values for more severe states, which can thus partially account for the greater range of index values for the EQ-5D [36].

The fact that utility scores appear to change over time, when patients report that their neck injury is the same, is evidence of potential response shift bias, where a patient’s subjective views and expectations change over time, causing a drift in the outcome score [37]. However, we have no evidence that this is more pronounced in one measure. There are specific tests available to assess whether this utility drift is actually the result of a response shift, rather than simply measurement error, such as a then-test, where patients are asked to retrospectively recall their health status (as they now perceive it) at a previous time point, and these are compared to the answers they gave at that time point itself [38]. However, such data were not available from the MINT study so no such test could be performed.

This study was helped by having access to a large cohort of patients with whiplash associated disorders, in contrast to many studies looking at the properties of instruments that have much smaller sample sizes. The collection of data at four separate time points is also an advantage over simply having two data points per individual. However, since the data used came from a clinical trial, all the usual caveats apply about the differences between trials and clinical practice, and the possibility for this to bias results, though since this was a pragmatic trial this should have less of an effect than in other situations [39]. Further, the lack of NDI data at baseline is a substantial limitation, making comparison between the NDI and other measures much more problematic than for those where we have contemporaneous data. There is also a concern due to the large amount of missing data in the study (less than 50% of participants returned questionnaires at all 4 time points), which could introduce bias. However, these response rates were similar to those for patient-reported outcome measures in other trials looking at whiplash interventions [40,41].

In conclusion, the evidence suggests that, for whiplash studies where only one generic health outcome measure is to be included, the EQ-5D is likely to offer advantages over the SF-12 and its preference-based derivative (SF-6D). Whilst this is the first study to look specifically at whiplash injuries, the finding that the EQ-5D and SF-6D do not provide interchangeable utility values, and that the EQ-5D is likely to have advantages over the SF-6D, is supported by other studies looking at neck injuries [42]. Comparisons with the NDI are more difficult, as there may be particular reasons for incorporating a condition-specific measure as opposed to a generic one in studies of whiplash associated disorders, whilst conversely the EQ-5D has the advantage of being preference-based, and can thus be used in cost-utility evaluations. Previous studies have shown the NDI to have good internal consistency, test-retest reliability and responsiveness [12,43]. Nevertheless, we found little evidence for better performance by the NDI when compared with the EQ-5D.

## Competing interests

The authors declare that they have no competing interests.

## Authors’ contributions

SP had the idea for the study, oversaw its design, contributed to the interpretation of the data, and redrafted the paper. JP did all the analyses, interpreted the results, and drafted the paper. SL, MW and EW assisted in the design of the study, interpretation of results and discussion of the findings. All authors read and approved the final manuscript.
